# Exposure of Primate Reservoir Hosts to Mosquito Vectors in Malaysian Borneo

**DOI:** 10.1007/s10393-022-01586-8

**Published:** 2022-05-13

**Authors:** Rebecca Brown, Milena Salgado-Lynn, Amaziasizamoria Jumail, Cyrlen Jalius, Tock-Hing Chua, Indra Vythilingam, Heather M. Ferguson

**Affiliations:** 1 Department of Vector Biology, Liverpool School of Tropical Medicine and Hygiene, Liverpool, L3 5QA UK; 2 Danau Girang Field Centre C/O Sabah Wildlife Department, Wisma Muis, Kota Kinabalu, Sabah, Malaysia; 3 School of Biosciences and Sustainable Places Research Institute, Cardiff University, Cardiff, UK; 4 Wildlife Health, Genetic and Forensic Laboratory, Kampung Potuki, Kota Kinabalu, Sabah Malaysia; 5 Department of Pathobiology and Medical Diagnostics, Faculty of Medicine and Health Sciences, Universiti Malaysia Sabah, Kota Kinabalu, Sabah Malaysia; 6 Department of Parasitology, Faculty of Medicine, University of Malaya, Kuala Lumpur, Malaysia; 7 Institute of Biodiversity, Animal Health and Comparative Medicine, College of Medical, Veterinary and Life Sciences, University of Glasgow, Graham Kerr Building, University Avenue, Glasgow, G12 8QQ UK

**Keywords:** *Plasmodium knowlesi*, Zoonotic malaria, Vector-borne disease, *Anopheles balabacensis*, *Macaca fasicularis*, Mosquito magnet independence traps

## Abstract

Several vector-borne pathogens of primates have potential for human spillover. An example is the simian malaria *Plasmodium knowlesi* which is now a major public health problem in Malaysia. Characterization of exposure to mosquito vectors is essential for assessment of the force of infection within wild simian populations, however few methods exist to do so. Here we demonstrate the use of thermal imaging and mosquito magnet independence traps (MMIT) to assess the abundance, diversity and infection rates in mosquitoes host seeking near long-tailed macaque (*Macaca fasicularis*) sleeping sites in the Lower Kinabatangan Wildlife Sanctuary, Malaysian Borneo. The primary *Plasmodium knowlesi* vector, *Anopheles balabacensis*, was trapped at higher abundance near sleeping sites than control trees. Although none of the *An. balabacensis* collected (*n* = 15) were positive for *P. knowlesi* by PCR screening, two were infected with another simian malaria *Plasmodium inui*. Analysis of macaque stools from sleeping sites confirmed a high prevalence of *Plasmodium* infection, suspected to be *P. inui*. Recently, natural transmission of *P. inui* has been detected in humans and *An. cracens* in Peninsular Malaysia. The presence of *P. inui* in *An. balabacensis* here and previously in human-biting collections highlight its potential for spillover from macaques to humans in Sabah. We advocate the use of MMITs for non-invasive sampling of mosquito vectors that host seek on wild simian populations.

**Supplementary Information:**

The online version contains supplementary material available at 10.1007/s10393-022-01586-8.

## Introduction

Non-human primates (NHPs) are reservoirs of vector-borne pathogens that can infect humans. Some already pose significant public health problems, such as the simian malaria parasites *Plasmodium simium* (Brasil et al. 2017) and *P. brasilianum* (Lalremruata et al. 2015) in South America. Several human vector-borne diseases have sylvatic origins, including Yellow Fever, Zika and Dengue (Rodhain 1991; Vorou 2016) alongside other lesser known viruses with potential to emerge in humans (Valentine et al. 2019). Understanding the force of infection in wild simian populations is crucial for assessment of potential for human spillovers, and the possibility of disrupting transmission in simian populations to mitigate against this. Such assessment requires estimating simian exposure to mosquito vectors, however there are few practical methods to do this. Current methods are invasive and rely on captive monkeys used as baits in traps, and may not reflect exposure in a natural population. There would be great value in finding a non-invasive and representative method for characterizing simian exposure to mosquito vectors.

One of the most notable simian vector-borne diseases (VBD) of public health significance in Southeast Asia is *Plasmodium knowlesi,* a zoonotic malaria whose natural hosts are long-tailed and pig-tailed macaques and *Presbytis* leaf-monkeys and is transmitted by Leucosphyrus group *Anopheles* (Knowles 1932; Wharton et al. 1963; Warren and Wharton 1963; Jeyaprakasam et al. 2014). Since the first cluster of human cases was detected in 2004 (Singh et al. 2004), *P. knowlesi* has become the most common cause of malaria in people in Malaysian Borneo (Hussin et al. 2013). In 2014, human cases of another macaque malaria, *P. cynomolgi,* were also reported in Malaysia (Ta et al. 2014; Law 2018). Other macaque malarias (*P. coatneyi*, *P. fieldi* and *P. inui* (Wong et al. 2015a; Manin et al. 2016)) have been detected in mosquito vectors in Malaysian Borneo, and recently *P. coatneyi* and *P. inui* were found infecting humans in Malaysia (Yap et al. 2021; Liew et al. 2021). Additional VBDs circulate in simian species in Malaysia that can infect people e.g. sylvatic Dengue in macaques, leaf monkeys and orangutans (Valentine et al. 2019; Rossi et al. 2012; Young et al. 2017), and the filarial worm *Brugia malayi* in leaf monkeys (Kwa 2008; Cheong et al. 1984). This wide range of potential VBDs necessitates surveillance of vectors biting simians to provide information on their abundance and infection prevalence to evaluate the infection or spillover risk posed to humans.

Characterization of VBD transmission in simians has been hindered by logistical and ethical constraints. To date there are a limited range of tools for studying simian exposure to VBDs; most being invasive by requiring blood sampling (Martinelli and Culleton 2018; Deane 1967; Dissanaike 1910). Alternative non-invasive methods for detecting malaria parasite DNA in faecal samples (Liu et al. 2010; Mapua et al. 2016; Nys et al. 2013; Assis et al. 2016; Abkallo et al. 2014; Siregar et al. 2015; Kawai et al. 2014) are promising but are yet to be widely applied and optimized. Similar constraints apply to assessment of simian exposure to mosquito vectors. This has generally been conducted through “Baited Traps” in which monkeys are placed in cages inside a net with gaps to allow mosquitoes attracted to enter but not leave (Wharton et al. 1963; Tan et al. 2008; Vythilingam et al. 2008; Jiram et al. 2012). Contemporary animal welfare regulations for working with captive monkeys often make such approaches unfeasible. Alternative less invasive approaches such as “e-nets” in which macaques are held in larger cages and have their odour collected and channelled to attract mosquitoes are logistically challenging and yield few vectors (Hawkes et al. 2017). Finally, all methods that require the use of a host ‘bait’ require capture of wild monkeys or handling of captive individuals; both of which are invasive and could cause distress. Identification of less invasive methods for sampling the vector population that host seeks on wild simian populations would be of great value.

So far most investigation of the mosquito vectors of macaque VBDs have been conducted in areas near human settlements (Tan et al. 2008; Vythilingam et al. 2008; Jiram et al. 2012; Hawkes et al. 2017) which may not be reflective of natural transmission cycles within simian populations in the absence of humans. Characterisation of natural cycles of simian malaria transmission in habitats with less human disturbance could help predict future spillover risk to humans. Identification of the vectors responsible for transmission and the species of parasites they carry will provide information on potential spillover risk to humans following encroachment on a formerly undisturbed habitat. Further to identification of future spillover risks, this will allow assessment of the feasibility of disrupting transmission in simian reservoir populations.

Here we evaluated the use of commercially available Mosquito Magnet Independence Traps (MMIT) to passively sample malaria vectors host seeking in the vicinity of long-tailed macaques within the Lower Kinabatangan Wildlife Sanctuary (LKWS) Sabah, Malaysia. Aims were to assess the performance of the MMIT in terms of the abundance and diversity of potential vector species captured near macaque roosts versus uninhabited trees, and whether infection rates in vectors caught near roost sites were reflective of infection prevalence in macaques as assessed from faecal samples. We also investigated relationships between malaria vector abundance in MMITs, macaque troop size as measured with thermal imagery, and environmental factors. Whilst Mosquito Magnet Traps have been investigated for passive surveillance of human malaria vectors (Hiwat et al. 2011a, 2011b; Sant’Ana et al 2014; Xue et al. 2010; Chaves et al. 2014; Vezenegho et al. 2014; Li et al. 2010), to our knowledge this is the first time they have been evaluated in a wild simian population.

## Methods

### Study Site

**Figure 1:**
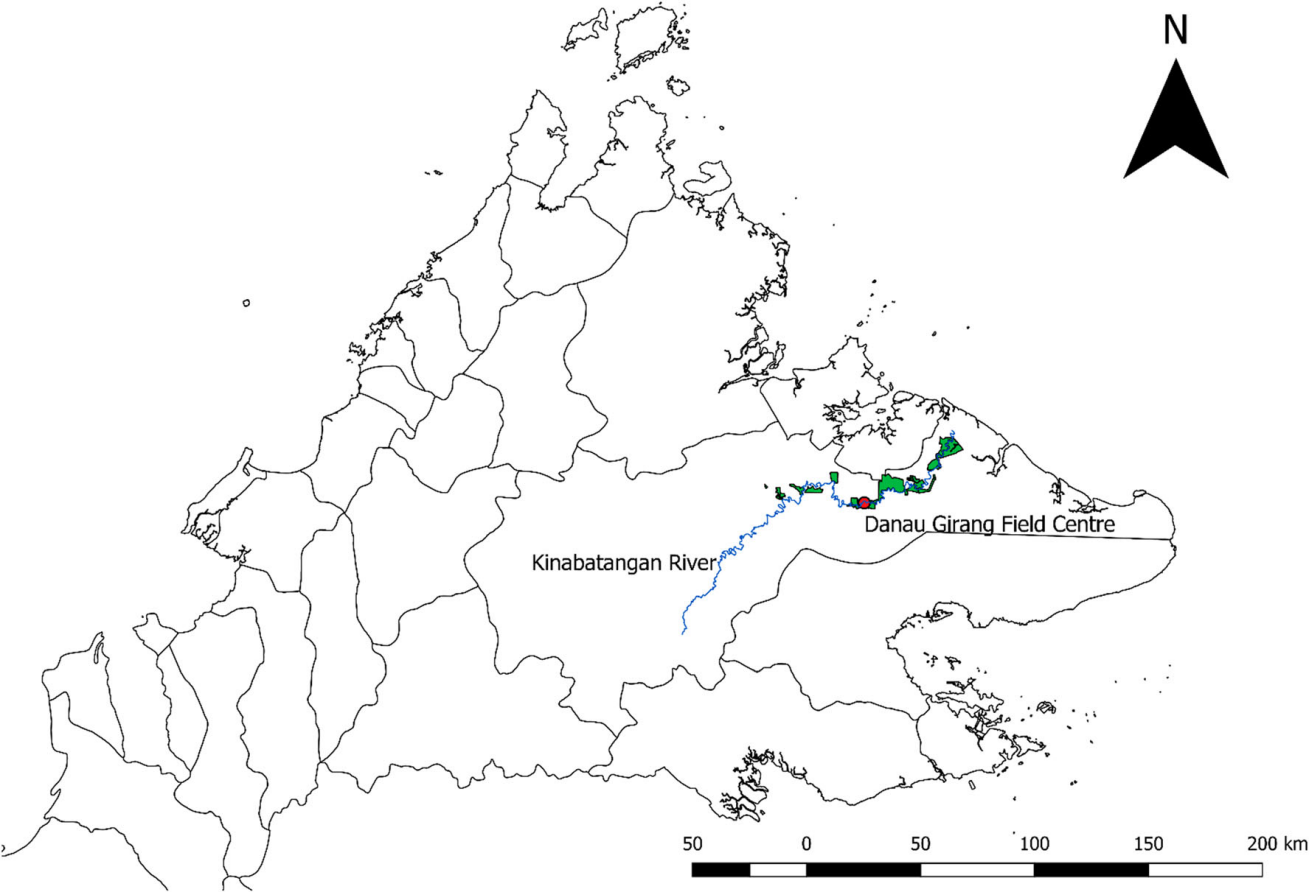
Map of Sabah indicating the location of the Danau Girang Field Centre (red) along the Kinabatangan River (blue). Green areas indicate boundaries of the Lower Kinabatangan Wildlife Sanctuary (Lots 1–10) and black lines show administrative districts.

This study was conducted at the Danau Girang Field Centre (DGFC), Lot 6 of the LKWS (5°24′49.93" N, 118°02′18.58" E) (Fig. 1). The LKWS is a protected secondary disturbed forest area (ranging from 10 to 60 years old) that contains primary to secondary lowland dipterocarp forest, mangrove and oil palm plantations (Hing 2012; Boonratana 1994). The sanctuary spans 27,000 ha (Estes et al. 2012), and hosts ten primate species including the two reservoir hosts of *P. knowlesi:* long-tailed macaques (*Macaca fasicularis*), and pig-tailed macaques (*Macaca nemestrina*). In 2002, population densities (per km2) were estimated as 16.82 for *M. fasicularis* and 3.30 for *M. nemestrina* (Hiwat et al. 2011a). The nearest human settlement is at least 15 km downstream from DGFC.

### HLC vs MMIT Trap Comparison

A pilot study was performed to confirm the MMIT (Mosquito Magnet, model: MM3200, supplier: Syarikat Thiam Siong Sdn Bhd, Sabah) was capable of sampling *Anopheles* in this environment (Fig. S1). The MMIT was compared with the standard Human landing catch (HLC) method; which is known to be efficient for sampling the *P. knowlesi* vector *An. balabacensis* (Wong et al. 2015a; Tan et al. 2008; Jiram et al. 2012). The MMIT lures mosquitoes using mammalian odour bait (CO_2_ and octenol), heat and water vapour (Sant’Ana et al 2014; Vezenegho et al. 2014). The MMIT was modified to run off batteries (4 × 1.5 V) and on locally available gas (30% propane: 70% butane).

Each night, one HLC and one MMIT site were selected; with stations ~ 100 m apart on one of three walking trails (Fig. S2). The following night the HLC and MMIT switched sites in a cross over design. This was repeated for ten nights of collections. Hourly collections were conducted from 18:00 – 00:00 h to coincide with the peak biting time of *An. balabacensis* (18:00—20:00 h (Wong et al. 2015a; Vythilingam et al. 2005)). One person performed HLC accompanied by an assistant. The same individuals had the role of collector and assistant for the duration of the study. Each hour comprised 45 min of trapping and 15 min break. The MMIT was switched off during the break and the collection net replaced.

### Use of MMIT for Sampling Vectors Near Macaque Sleeping Sites

Mosquito sampling using MMITs was conducted along a 20 km section of the Kinabatangan river (560 km, Fig. 1). Trees (*Colona*, *Nauclea subdita, Pterospermum acerifolium*, *Kleinhovia hospita* and *Ficus*) of a minimum 20 m depth lining the river bank (Stark et al. 2018) are used as sleeping sites for several simian species (Matsuda et al. 2016; Goossens et al. 2002), including long-tailed macaques. The study site was divided into ten 2 km transects (Fig. S3). The home range of long-tailed macaques in this reserve was estimated as 1.25 km^2^ in a previous survey (Goossens et al. 2002). To avoid repeated sampling near the same macaque troop, sampling was conducted in different 2 km transects each night. Mosquito sampling took place in each transect once every ten nights; with the transect selected randomly using the Random UX app. Sampling was conducted for five nights consecutively then a one night break; resulting in 38 sampling nights between September and November of 2017. Each transect was sampled 3–4 times during this period, with traps placed on alternate sides of the river on each visit.

Upon arrival at the selected transect (17:30 h), the river banks were scanned with a thermal imaging camera to identify potential macaque troops by driving slowly up and down the river. When the camera indicated presence of a troop, binoculars were used to inspect trees for long-tailed macaques. Once the presence of roosting macaques was confirmed, a MMIT was placed near the bottom of their sleeping tree (conditional on bank being accessible, Fig. S4). Macaques generally moved from the selected tree to higher up in the canopy or deeper inside the forest as the boat approached, but would return after the trap was placed. There was only one instance of macaques absent in the morning, we think because of disturbance from a nearby plantation where the wildlife corridor narrowed. A second MMIT was placed at least 100 m away at a ‘control’ tree that was of similar structure and species, but uninhabited for that evening by macaques or other monkeys. This ‘control’ tree enabled differentiation of mosquitoes specifically host seeking in the vicinity of macaques. Trees lining the riverbank were used for sampling, however the exact distance of trees from the river was not recorded. The width and gradient of the bank between the river and the forest edge varied but trees were selected based primarily on ease of access from the boat combined with either the presence of macaques or species of tree matching that of the selected macaque tree. Thus trees on the fringe of the forest patch were selected and deep penetration of the forest patch was not performed.

Mosquitoes were collected at macaque sleeping and control sites each night from 18:00 to 06:00 h. Before sunrise and movement of macaques (approximately 05:30 h), the number of macaques sleeping in the tree where the MMIT was placed was counted from the boat using the thermal camera. Daily rainfall data (collected by rain gauge) was provided by DGFC.

### Mosquito Processing

Mosquitoes were stored at − 20 ˚C for approximately 12 h then identified to genera and species where possible (Rattanarithikul et al. 2005; Rattanarithikul et al. 2005; Rattanarithikul et al. 2006 Rattanarithikul et al. 2006). Leucosphyrus group *Anopheles* were identified using Sallum et al. (2005). All identified mosquitoes were stored in 95% ethanol. Molecular analysis was performed on Leucosphyrus group *Anopheles*, *An. barbirostris gp. (An. barbirostris and An. donaldi), An. epiroticus and An. tesselatus* (malaria vectors in Sabah or elsewhere in SE Asia, (Vythilingam et al. 2005; Rattanarithikul et al. 2006; Rahman et al. 1993; Hawkes et al. 2019; Sriwichai et al. 2016; Manguin et al. 2008)) to screen for *Plasmodium* infections using the method described in (Brown et al. 2008).

### Macaque Faecal Collection

Each morning after emptying MMIT traps, the ground within a 20 m radius of sleeping trees was inspected for the presence of fresh macaque stools (see Supplementary Methods S1). Stool samples were homogenized in RNAlater solution then stored at  − 20 °C.

DNA was extracted from 200 µl of each stool solution using the QIAamp DNA Stool Mini Kit. DNA was eluted in 100 µl buffer AE and stored at  − 20 °C. Samples were screened by PCR for detection of DNA from the *Plasmodium* genus (see Supplementary Methods S2). *Plasmodium* positive samples were then screened to test for the specific presence of *P. knowlesi* following the method of Kawai et al. (2014)*.*

### Statistical Analysis

Data were analysed using the R statistical programming software (3.4.2) with packages lme4 and multcomp (Bates et al. 2015; Hothorn and Bretz 2008). Generalized Linear Mixed Models (GLMMs) were used to compare the abundance of mosquitoes in HLC and MMIT; with comparisons made for all mosquitoes and just *Anopheles*. Negative binomial GLMMs were used to account for overdispersion in mosquito count data (Lindén and Mäntyniemi 2011). The response variable was the abundance of (i) all mosquitoes (ii) *Anopheles* per night. The main fixed effect was trap type with random effects fit for date and trail. A post hoc Tukeys’ test was used to assess differences in mosquito abundances between traps. The vegan package (Oksanen et al. 2020) was used to measure *Anopheles* diversity in HLC and MMIT catches. Four diversity indices were calculated: species richness, rarefied species richness, Simpson’s index and the Shannon index (Brown et al. 2008).

Sampling of mosquitoes near trees where macaques were sleeping was conducted for 38 nights. On a few occasions, macaques or other monkeys were present at the control site in the mornings or the traps stopped working overnight due to failure of gas supply or batteries. Excluding these scenarios, data were available from 33 nights of sampling at control trees and 34 nights at trees with sleeping macaques. With this data, GLMMs were constructed to test for differences in *Anopheles* abundance between macaque sleeping sites and control trees. A negative binomial distribution was used with date set as a random effect. Negative binomial GLMs were used to test for differences in *An. balabacensis* abundance and *An. donaldi* abundance. Models tested for associations between mosquito abundance and macaque presence and abundance, and rainfall on the day of sampling. The significance of each variable was tested by backward elimination using likelihood ratio tests. Post hoc Tukey’s tests were performed to assess differences in mosquito abundance between sleeping site and control collections.

## Results

### HLC vs MMIT Trap Comparison

Overall, 2895 mosquitoes were collected in the HLC/MMIT trap comparison. Both HLC and MMITs collected mosquitoes belonging to the same eight genera (Table S1). Mosquitoes were identified to species level where possible, however due to time constraints, priority was given to *Anopheles*, *Culex* and *Mansonia*. *Aedes* and *Uranotaenia* mosquitoes were mostly identified to subgenus. In general, mosquitoes trapped by HLC were in better condition for morphological identification than those trapped in the MMIT because key characteristics necessary for species determination such as hairs and scales were better preserved.

Almost all *Anopheles* caught in the HLC could be speciated, except one individual that was missing features to distinguish between *An. barbirostris* or *An. donaldi*. Two *Anopheles* from MMIT collections (3.2% of total) could not be placed to a subgenus. Five *Anopheles* species were collected by HLC compared to 8 species with MMIT (Table 1). *Anopheles* diversity was higher in MMIT than HLC collections (Table 2). Both methods trapped the *P. knowlesi* vectors *An. balabacensis* and *An. donaldi*; with a higher proportion of these being caught by HLC (80.5%, *n* = 29) than MMIT (72.6%, *n* = 45) however this difference was not statistically significant (*P* = 0.37).

**Table 1:** *Anopheles* mosquitoes caught by Mosquito Magnet Independence Traps (MMIT) and Human Landing Catch (HLC) over ten nights of trap comparison study in Lower Kinabatangan Wildlife Sanctuary, Sabah

	Human-landing catch (HLC)	Mosquito Magnet Independence Traps (MMIT)
***Anopheles***	**36**	**62**
*An. balabacensis*	2	5
*An. barbirostris*	0	2
*An. barbirostris/donaldi*	1	5
*An. barbumbrosus*	0	2
*An. cellia* subgenus	0	1
*An. donaldi*	27	40
*An. kochi*	0	1
*An. montanus*	2	1
*An. roperi*	1	1
*An. tesselatus*	3	2
Unknown *Anopheles* spp.	0	2

**Table 2:** Measures of diversity in *Anopheles* species from Mosquito Magnet Independence Traps (MMIT) and Human Landing Catch (HLC) collections from a ten-night trap comparison study in Lower Kinabatangan Wildlife Sanctuary, Sabah

Trap Type	Abundance	Species richness	Rarefied species richness	Shannon Index	Simpson’s Index
HLC	35	5	2.93	0.84	0.39
MMIT	54	8	3.23	1.03	0.44

**Figure 2:**
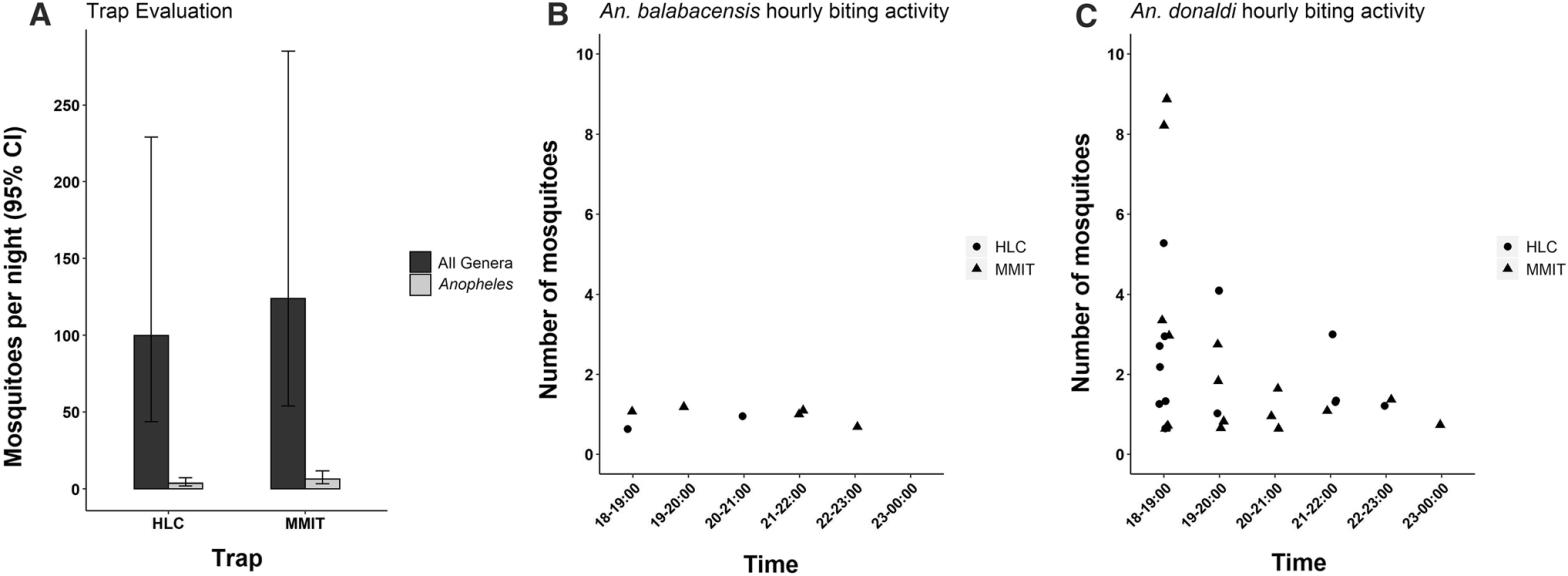
A) Mean abundance of mosquitoes caught per night by Human Landing Catch (HLC) and Mosquito Magnet Independence Traps (MMIT) as predicted by negative binomial generalized linear mixed models (GLMM). Error bars represent 95% confidence intervals B) *An. balabacensis* and C) *An. donaldi* trapped per hour by human-landing catch (HLC) and mosquito magnet independence traps (MMIT).

Although mosquito numbers tended to be higher in MMIT than HLC collections, the mean nightly abundance was not significantly different (Tukey’s test: *P* = 0.39, Fig. 2A). The GLMM to explore *Anopheles* abundance between trap types failed to converge, so a negative binomial GLM without random effects was used instead. This showed that the mean nightly abundance of *Anopheles* did not vary between trapping methods (Tukey’s test: *P* = 0.210, Fig. 2A). *Anopheles donaldi* was most abundant between 18:00 and 20:00 h (Fig. 2C), whereas *An. balabacensis* biting rates were relatively constant between 18:00 and 23:00 h with none collected between 23:00 and24:00 h (Fig. 2B).

### MMIT to Sample *Anopheles* Host Seeking Near Macaques

Overall, 11,400 mosquitoes from eight genera were collected in MMITs placed near macaque sleeping sites and control trees (Table S2). Both malaria vector species, *An. balabacensis* and *An. donaldi* were trapped at sleeping sites and control trees. Mansonia spp. (*Ma. uniformis, Ma. bonneae, Ma. dives, Ma. indiana, Ma. annulata* and *Ma. annulifera)* vectors of *Brugia malayi* filariasis were collected in high abundance (Table S2) and were distributed evenly between ‘sleeping site’ and ‘control’ catches.

Combining over all species in the genera, the mean nightly abundance of *Anopheles spp.* was not significantly associated with the presence (X^2^ = 0.23, df = 1, *P* = 0.62) or number of macaques (X^2^ = 0.84, df = 1, *P* = 0.35) at a tree, or with daily rainfall (X^2^ = 0.50, df = 1, *P* = 0.47) (Fig. S5).

**Figure 3:**
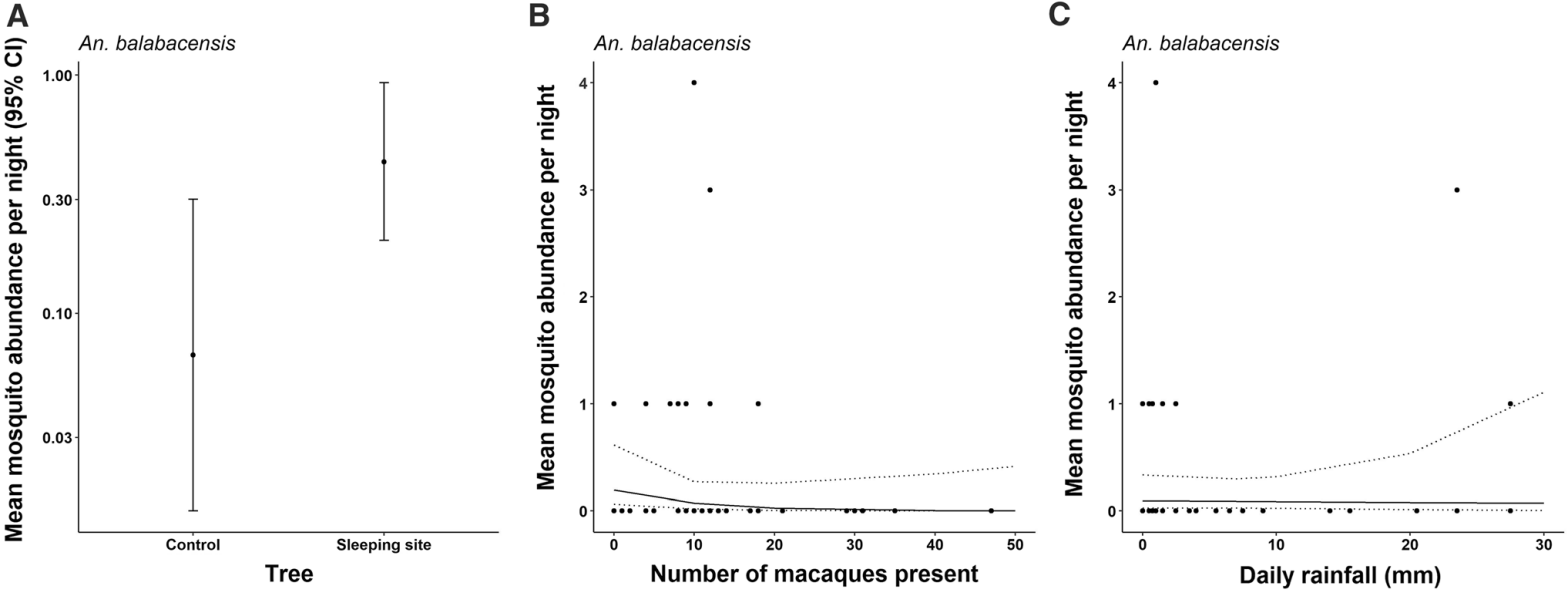
Influence of A) macaque presence/absence, B) number of macaques present and C) daily rainfall on the mean nightly *An. balabacensis* abundance collected by Mosquito Magnet Independence Traps (MMIT). Points indicate observed data in B and C, with the line indicating the predicted association. Error bars and dashed lines are 95% confidence intervals.

The simian malaria vector *An. balabacensis*, however, was significantly impacted by the presence of macaques at sampling sites. The mean abundance of *An. balabacensis* was significantly higher near macaque roost sites than at control trees (LR stat = 7.83, Df = 1, *P* < 0.01, Fig. 3A), but was not related to number present (LR stat = 2.10, Df = 1, *P* = 0.15) or daily rainfall (LR stat = 0.845, Df = 1, *P* = 0.36, Fig. 3B and C).

The total number of *An. donaldi* caught at macaque sleeping sites (n = 106) was lower than at control trees (n = 211, Table 3); however this difference was not statistically significant (Tukeys: *P* = 0.43). The abundance of *An. donaldi* was not dependent on the presence or absence of macaques (LR stat = 0.59, Df = 1, *P* = 0.44), the number of macaques present (LR stat = 0.62, Df = 1, *P* = 0.43) or the daily rainfall (LR stat = 0.55, Df = 1, *P* = 0.46) (Figure S6).

**Table 3:** *Anopheles* mosquitoes caught with Mosquito Magnet Independence Traps (MMIT) at trees with and without sleeping macaques (control trees) within the Lower Kinabatangan Wildlife Sanctuary, Sabah

	Macaque sleeping sites (34 nights)	Control trees (33 nights)
***Anopheles***	**250**	**476**
*An. balabacensis*	13	2
*Barbirostris gp*	122	251
*An. barbirostris*	0	2
*An. donaldi*	106	211
*An. epiroticus*	1	0
*An. gigas*	1	0
*An. montanus*	2	2
*An. roperi*	0	1
*An. tesselatus*	2	0
*An. umbrosus gp*	1	2
Unknown *Anopheles* spp.	2	5

### Plasmodium Infections in Mosquitoes and Macaque Stools

Eighty-one *Anopheles* collected in the initial HLC versus MMIT trap comparison were tested for malaria (*An. donaldi* = 61, *An. balabacensis* = 7, *An. barbirostris/donaldi* = 5, *An. tesselatus* = 5, *An. celia* group = 1, *An.* unknown = 2). Of these, one tested positive for *Plasmodium* infection (n = 1/81). In the larger study using MMITs at macaque sleeping sites and control trees, 398 *Anopheles* were tested for malaria (Barbirostris group = 373 (including *An. barbirostris* (2) and *An. donaldi* (317)), *An. balabacensis* = 15, *An. epiroticus* = 1, *An. tesselatus* = 2 and unidenitifed *Anopheles* species = 7, Table 3). Of these, one tested positive for *Plasmodium* (n = 1/398)*.* Both infections were confirmed to be *P. inui*. Both infections were detected in *An. balabacensis*, representing an overall infection rate of 9% (n = 2/22) in this vector species.

Of the 46 long-tailed macaque faecal samples collected, 17 (37%) tested positive for *Plasmodium*. However in the subsequent round of PCR analysis to test for *P. knowlesi*, none were positive. Samples were not screened for other malaria species, thus the identity of *Plasmodium* infections remains unknown.

## Discussion and Conclusions

This study to our knowledge provides the first evaluation of the use of MMIT for sampling the mosquitoes that host seek on wild simians. We show that the abundance of all mosquitoes (pooled across genera), and *Anopheles* in particular was similar in collections made by MMITs and the HLC gold standard method. The MMIT and HLC caught mosquitoes from the same genera however the MMIT caught a greater diversity of *Anopheles* species than HLC. Our results confirm that the MMIT can be used as an indirect exposure-free alternative to the HLC. MMITs placed below macaque roosting trees collected several known vectors of zoonotic and human malaria (*An. balabacensis*, *An. donaldi* and *An. barbirostris*). While *Anopheles* density was not higher overall at trees with than without macaques, the abundance of the confirmed primate vector *An. balabacensis* was significantly higher near macaque sleeping sites. This implies *An. balabacensis* were actively host seeking on macaques. Analysis of macaque faecal samples indicating a high prevalence of *Plasmodium* infection (37%). However, the zoonotic malaria *P. knowlesi* was not detected in either vector or macaque samples here suggesting transmission was primarily of other simian parasite species. Mosquito infections were confirmed to be *P. inui*; a simian malaria parasite that was recently found naturally infecting humans in Malaysia (Yap et al. 2021; Liew et al. 2021). Despite the absence of *P. knowlesi*, the ability of MMITs to detect known vector species feeding at macaque sleeping sites highlights its value for non-invasive monitoring of simian exposure to mosquito vectors. This tool could thus provide opportunity to study the transmission of *P. knowlesi* as well as other simian VBDs in wild monkey populations.

The abundance of *Anopheles spp.* was similar in MMIT and HLC collections, although the MMIT caught more species (8 vs 5 in the HLC). The greater diversity of Anophelines in the MMIT compared to HLCs, also seen in a Venezuelan setting (Rubio-Palis et al. 2013) may be due to the use of a general R-octenol bait that attracts both anthropophilc and zoophilic mosquitoes (Dekel et al. 2016), and/or that it releases a higher concentration of host cues than a single human collector. Here only one individual performed HLC, but it is well known that volatile emissions vary between people (Fenske and Paulson 1999). Further investigation using multiple participants in HLC is required for more robust evaluation of the relative performance of MMIT and HLC methods. However, given the MMIT has an advantage of enabling passive sampling and collected just as many *Anopheles* as HLC, it may be a more practical and ethically acceptable approach for sampling of malaria vectors host seeking on wild simians.

Despite its success here, there are several points to consider before selecting the MMIT as a research tool. It has a high initial cost however this may be equivalent to/less than the cost of hiring staff to perform HLC (Vezenegho et al. 2015), therefore the duration of the study will impact the choice of method. The MMIT is limited by high battery consumption and the local availability of gas refill. It is bulky, difficult to transport for long distances in jungle terrain and is not suitable for canopy installation or in areas of high elevation or steep slopes (Chaves et al. 2014). Other options exist such as the CDC light traps and BG sentinel traps however trap evaluation experiments demonstrate none catch a higher abundance and diversity of *Anopheles* than Mosquito Magnet Traps (Hiwat et al. 2011a; Brown et al. 2008; Dusfour et al. 2010).

The primary *P. knowlesi* vector in Sabah, *An. balabacensis*, was detected at significantly higher numbers near trees with than without macaques; indicating this species is an acceptable host type. This finding is in line with a host choice study involving baited electrocuting nets where *An. balabacensis* were lured towards odour cues emanating from either monkey or human hosts (Hawkes et al. 2017). However, there was no significant relationship between *An. balabacensis* abundance and the number of macaques at the sleeping site. Macaque troop size varied across sampling nights from 2 to 47 individuals (average ~ 14), thus incorporating substantial variability for detecting an association with mosquito density. Studies on malaria vectors have detected correlations (positive and negative depending on vector species) between adult Anopheline density and the density of humans (McCann et al. 2017; Kaindoa et al. 2016). The lack of association here, however may be the result of the odour plume of even one macaque being sufficient to lure *An. balabacensis*. Alternatively, mosquitoes could be attracted to the trees themselves. Macaques are known to revisit sleeping trees (Goossens and Ambu 2012) thus macaque odour cues could build up around a site, signalling a reliable bloodmeal source for vectors. Additionally, there could be environmental characteristics not measured here that contributed to higher *An. balabacensis* abundances at macaque sleeping sites.

Associations between *Anopheles* abundance in MMIT collections and rainfall and temperature were not detected. Higher temperature and rainfall have been demonstrated to increase mosquito abundances in MMITs used in the Brazilian rainforest (Chaves et al. 2014). No association was detected between temperature or rainfall and *An. balabacensis*; but ability to test for this was limited by small sample sizes. To investigate seasonal fluctuations in vector abundance with rainfall and temperature, we recommend more intensive longitudinal sampling across a full year to increase sample sizes and capture the extremes of environmental variation.

The primary focus of this study was investigation of *P. knowlesi* transmission within its wildlife reservoir in the absence of humans. However, malaria infections were detected in only two *An. balabacensis* and in both cases it was *P. inui*. *Plasmodium inui* is commonly found in wild macaques (Collins et al. 2007) and can infect humans under experimental conditions (via blood transfusion or infected mosquito bites in the laboratory (Vythilingam et al. 2013). Natural human infections of *P. inui* as well as *P.cynomolgi, P. coatneyi* and *P. simiovale* have been recently detected in Peninsular Malaysia (Yap et al. 2021; Liew et al. 2021). Relatively high rates of *P. inui* and other simian malarias (*P. cynomolgi, P. fieldi and P. coatneyi*) have been described in *An. balabacensis* in village settings in Sabah (Manin et al. 2016). Thus, people are regularly exposed to these parasites in peri-domestic as well as forest settings; raising the possibility that *P. inui* could pose a significant risk for zoonotic spillover in Sabah in the future.

The absence of *P. knowlesi* infection in mosquito vectors was matched with its absence in the macaque population. Although *Plasmodium* DNA was detected in more than a third of macaque stool samples, none of these samples were identified as *P. knowlesi.* We hypothesise these infections were most likely *P. inui* based on its confirmation in the local *An. balabacensis* population. *Plasmodium knowlesi* prevalence in macaques has been reported at 6.9% and 30% in Peninsular Malaysia (Vythilingam et al. 2008; Akter et al. 2015), and 20% and 86.6% in Sarawak (2013) and Lee et al. (2011). However these estimates were derived from analysis of macaque blood samples which have greater sensitivity to detect low density infections than the faecal screening method used here (Loy et al. 2018). However, another study also based on analysis of macaque blood samples reported a much lower prevalence of *P. knowlesi* (0.4% (Zhang et al. 2016)) indicating macaque infection rates are naturally heterogeneous. It is often assumed that the force of *P. knowlesi* infection coming from macaques to humans is high throughout Sabah; given one study found *P. knowlesi* infection in 20% of the faeces collected from wild long-tailed macaques within the Kudat District, the hotspot of human infection in 2013–2014 (Salgado-Lynn, unpublished data). In the same study, 80% of the blood samples of macaques from a different district in Sabah were positive for *Plasmodium*, 66% of which were positive for *P. knowlesi*. However the apparent absence of *P. knowlesi* infection here indicates the force of infection may vary considerably between wild reservoir populations. Therefore, recent efforts to generate *P. knowlesi* risk maps based on macaque distribution (Loy et al. 2018; Zhang et al. 2016; Chua et al. 2017) may be limited by failure to incorporate underlying variation in infection prevalence within macaque populations. Furthermore, blanket control policies based on macaque culling may be both ethically questionable and have limited impact. Further monitoring is required through vector and macaque stool screening to track prevalence of malaria infection in different simian species to understand the variation between macaque populations and the resultant risk to neighbouring humans.

The findings from this study can provide more information on likely spillover routes between primates and humans in this setting, in terms of the vectors that may be implicated at different stages of the process. Here *An. balabacensis* was more abundant nearby sleeping macaques, whereas *An. donaldi* did not exhibit the same behaviour. Therefore it is possible that *An. balabacensis* is more specialized and could play a major role in macaque to macaque transmission as well as macaque to human transmission. *Anopheles balabacensis* has been widely implicated as the key vector in transmitting primate malaria to people in Sabah and has been detected feeding on humans in village, farm and forest settings (Wong et al. 2015a; Manin et al. 2016; Brown et al. 2008; Chua et al. 2017). *Anopheles donaldi* has been collected on humans in similar habitat types (Hawkes et al. 2019; Brown et al. 2008; Wong et al. 2015b) and was found positive with *P. knowlesi* and *P.cynomolgi* (Hawkes et al. 2019) however, it is unknown whether these were sporozoite or oocyst infections. *Anopheles donaldi* is also known to be zoophilic (Vythilingam et al. 2005), therefore more study is required to understand if *An. donaldi* plays a role in the transmission of simian malaria to humans.

Here we demonstrate the suitability of MMIT for sampling mosquitoes host seeking in the vicinity of macaques and advocate its use as a tool for monitoring vector borne pathogens circulating in wild simian populations. In addition to its use for investigation of vector ecology it is a reliable alternative to performing HLC to study vectors feeding on people and removes the need to expose volunteers to potentially infectious mosquito bites. With the recent detection of naturally acquired *P. inui* infections in Peninsular Malaysia, and with the detection of the parasite in *An. balabacensis* here and in collections nearby homes (Manin et al. 2016; Chua et al. 2017; Wong et al. 2015b) people are likely frequently being exposed to this parasite in Sabah. This warrants close surveillance to monitor for increasing spillover of *P. inui* into human populations in this setting.

## Supplementary Information

Below is the link to the electronic supplementary material.

Supplementary file1 (DOCX 2352 KB)

Supplementary file2 (DOCX 23 KB)

Supplementary file3 (DOCX 19 KB)

## Data Availability

Data supporting the results reported in this article can be found in the Harvard Dataverse repository: https://doi.org/10.7910/DVN/ZS4VRY.

## Abbreviations

HLC: Human landing catch
MMIT: Mosquito magnet independence trap
LKWS: Lower Kinabatangan Wildlife Sanctuary
GLMM: Generalized linear mixed models
