# Supplementary material for: Exposure of Primate Reservoir Hosts to Mosquito Vectors in Malaysian Borneo
Source: Ecohealth. 2022 May 13;19(2):233–45. doi: 10.1007/s10393-022-01586-8 (PMC9276546; doi:10.1007/s10393-022-01586-8)

## List of Supplementary Files

Figure S1. A Mosquito Magnet Independence Trap (MMIT, left) and a view of the mosquito collection net within the MMIT (right).

Figure S2. Map of forest trails surrounding the main building of Danau Girang Field Centre in the Lower Kinabatangan Wildlife Sanctuary. Boxes indicate the sites used on Ficus (wet lowland forest), Kingfisher (wet lowland forest) and Kayu Malam (dry lowland forest) walking trails for human-landing catch (HLC) and Mosquito Magnet Independence Trap (MMIT) evaluation of collecting *Anopheles*. Blue lines depict bodies of water.

Figure S3. The 20km stretch of the Kinabatangan River surrounding Danau Girang Field Centre where macaque roosting sites and control trees were selected for mosquito collection. Purple dots indicate the boundary of each 2km transect that could be randomly selected for mosquito sampling on each night.

Figure S4. The Mosquito Magnet Independence Trap (MMIT) in position on the riverbank at the base of a *Ficus* (fig) tree to be used by a long-tailed macaque troop as their overnight resting place.

Figure S5. Influence of A) macaque presence/absence, B) number of macaques present and C) daily rainfall on the mean nightly *Anopheles* abundance collected by Mosquito Magnet Independence Traps (MMIT). Points indicate observed data in B and C, with the line indicating the predicted association. Error bars and dashed lines are 95% confidence intervals.

Figure S6. Influence of A) macaque presence/absence, B) number of macaques present and C) daily rainfall on the mean nightly *An. donaldi* abundance collected by Mosquito Magnet Independence Traps (MMIT). Points indicate observed data in B and C, with the line indicating the predicted association. Error bars and dashed lines are 95% confidence intervals.

Table S1. Mosquitoes caught by Mosquito Magnet Independence Trap (MMIT) and human-landing catch (HLC) over ten nights of trap comparison study in Lower Kinabatangan Wildlife Sanctuary, Sabah.

Table S2. Mosquitoes caught with Mosquito Magnet Independence Trap (MMIT) at trees with and without sleeping macaques (control trees) within the Lower Kinabatangan Wildlife Sanctuary, Sabah.


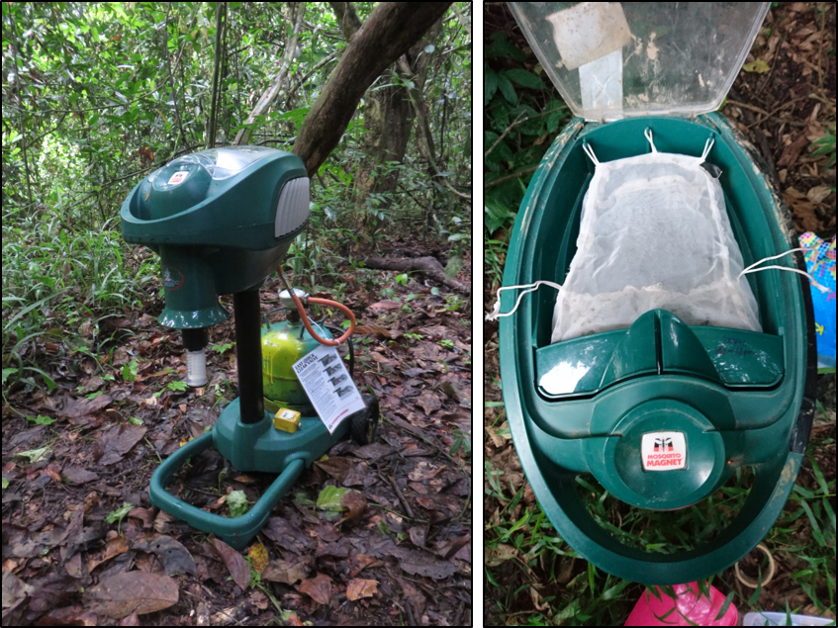


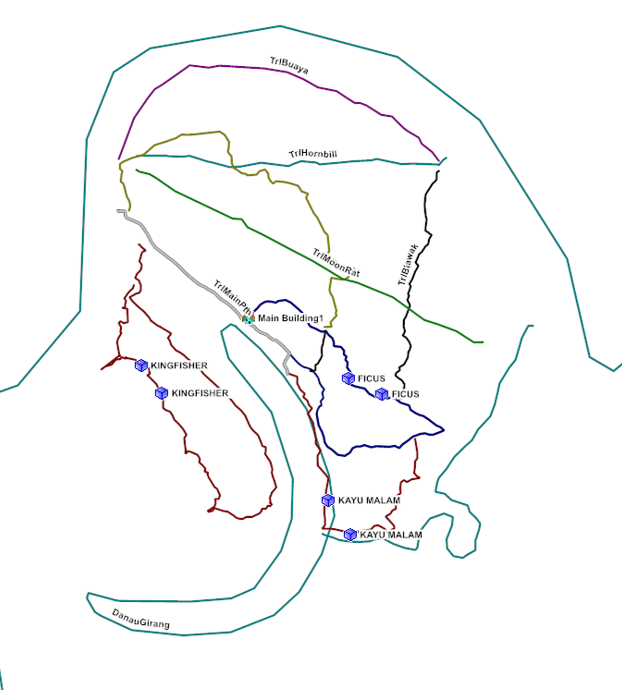


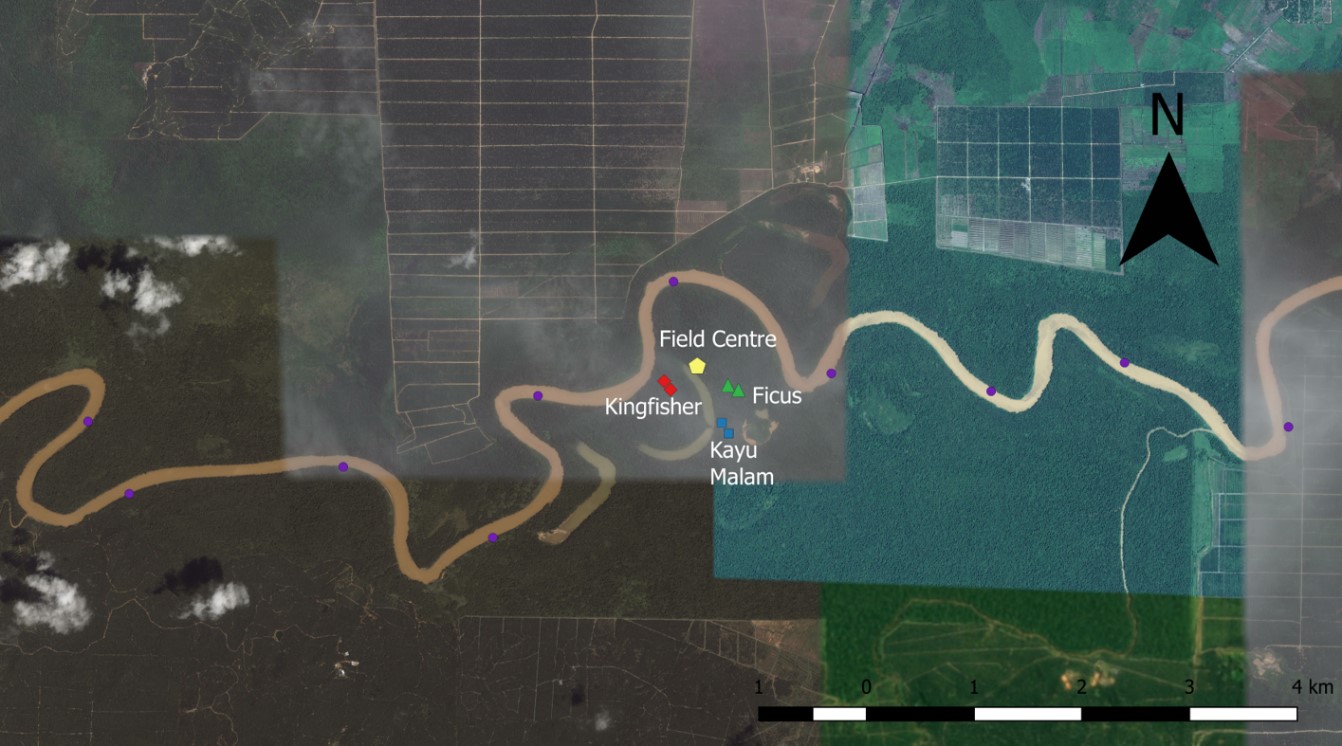


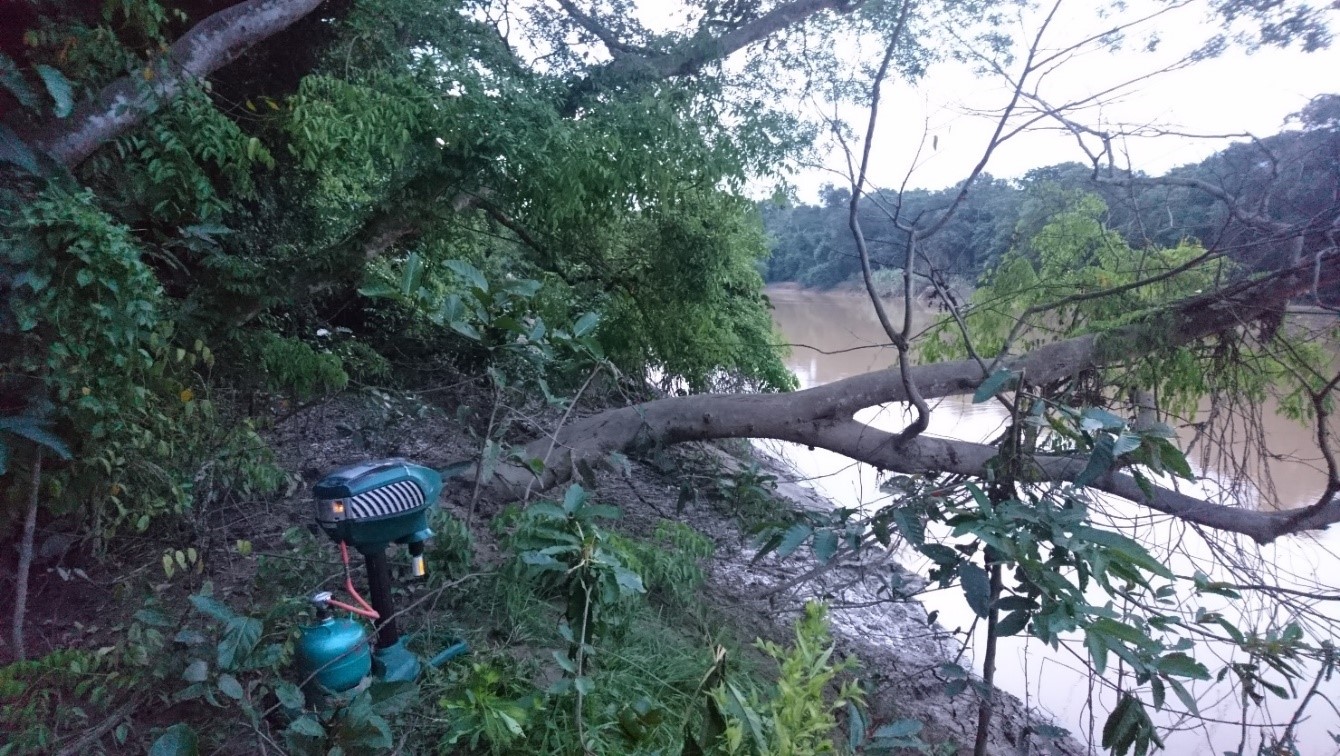


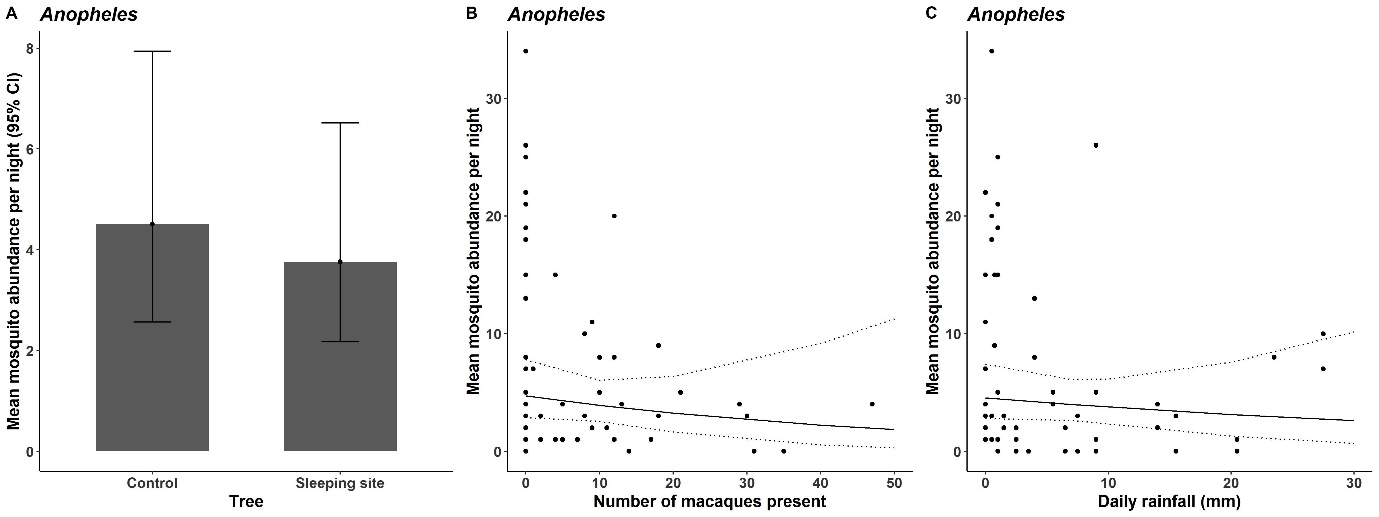


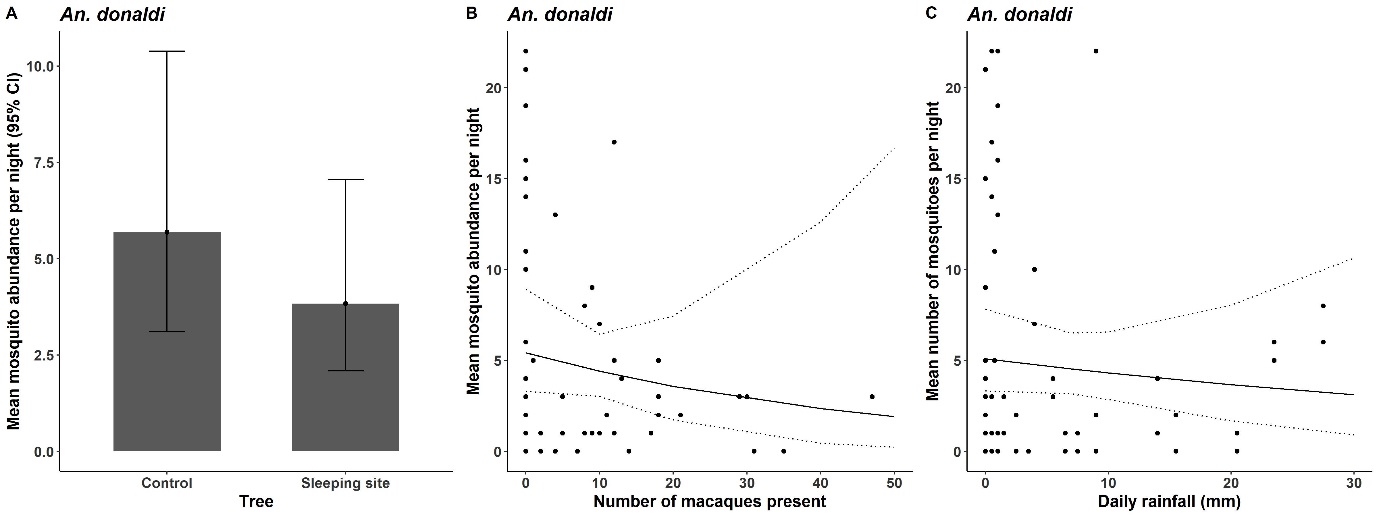

Supplement: Supplementary file 1 — Supplementary file1 (DOCX 2352 KB) [file 10393_2022_1586_MOESM1_ESM.docx]
